# Environmental factors explain spawning day deviation from full moon in the scleractinian coral *Acropora*

**DOI:** 10.1098/rsbl.2019.0760

**Published:** 2020-01-22

**Authors:** Yusuke Sakai, Masayuki Hatta, Seishiro Furukawa, Masakado Kawata, Naoto Ueno, Shinichiro Maruyama

**Affiliations:** 1 Division of Morphogenesis, National Institute for Basic Biology, Okazaki, Aichi, Japan; 2 Graduate School of Humanities and Sciences, Ochanomizu University, Bunkyo-ku, Tokyo, Japan; 3 Graduate School of Life Sciences, Tohoku University, Sendai, Miyagi, Japan; 4 Department of Basic Biology, School of Life Science, SOKENDAI (The Graduate University for Advanced Studies), Okazaki, Aichi, Japan

**Keywords:** coral reef, spawning synchrony, reproduction, *Acropora*, sea surface temperature, full moon

## Abstract

Broadcast-spawning scleractinian corals annually release their gametes with high levels of synchrony, both within and among species. However, the timing of spawning can vary inter-annually. In particular, the night of spawning relative to the full moon phase can vary considerably among years at some locations. Although multiple environmental factors can affect the night of spawning, their effects have not been quantitatively assessed at the multi-regional level. In this study, we analysed environmental factors that are potentially correlated with spawning day deviation, in relation to the full moon phase, in *Acropora* corals inhabiting seven reefs in Australia and Japan. We accordingly found that sea surface temperature and wind speed within one to two months prior to the full moon of the spawning month were strongly correlated with spawning day deviations. In addition, solar flux had a weak effect on the night of spawning. These findings indicate that *Acropora* have the capacity to adjust their development and physiology in response to environmental factors for fine-tuning the timing of synchronous spawning, thereby maximizing reproductive success and post-fertilization survival.

## 1. Introduction

Inter-annual variations in environmental conditions, e.g. temperature and photoperiod, can lead to shifts in the phase and amplitude of the seasonal oscillations of biological events. Scleractinian corals exhibit conspicuous spawning synchrony [1–3], which was first documented in several populations on the Great Barrier Reef, Australia, where the majority of corals spawn synchronously over a few evenings following the full moon phase in late spring or early summer [4]. However, although the spawning pattern of corals has become one of the most well-studied annual reproduction events, it remains unclear how these corals adjust to inter-annual variations in environmental factors.

The synchronous spawning of corals is plausibly regulated by successive environmental cues at multiple timescales—spawning month (or season), day and hour [5]. Although the month of spawning has been linked to a number of environmental factors, including temperature, rainfall, solar insolation and wind speed [6–9], an investigation assessing all these variables at the scale of the Indo-Pacific basin indicated that the peak month of spawning is most closely associated with seasonal rises in sea surface temperature (SST) [10]. The day of spawning is generally in tune with lunar periodicity, and assumed to be determined by variations associated with the lunar cycle, e.g. in moonlight, pressure or water motion [2]. For determining the spawning hour, the duration of darkness after sunset is reportedly a potential proximate cue [11,12].

Despite the high synchrony of spawning that occurs each year, there remains substantial inter-annual variation. In particular, the night of spawning in relation to the full moon phase, which is critical for spawning synchrony, has been shown to exhibit inter-annual variation at some sites. Several local-scale studies have indicated that high SSTs in the months prior to spawning are correlated with the lunar spawning day [13–16]. However, how and to what extent environmental factors influence inter-annual variations in the night of spawning have not been explored. To address this issue, we generated statistical models to analyse the multi-year-long and multi-regional data of the spawning days of the coral genus *Acropora*, the species of which are dominant in most shallow-water coral assemblages throughout the Indo-Pacific Ocean [10].

## 2. Material and methods

### (a) Multi-year-long dataset of spawning days in *Acropora* species

**Figure 1.**
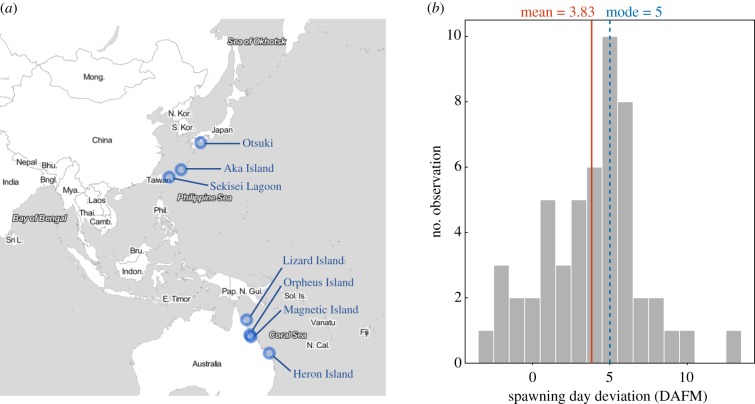
Location of the coral reefs and spawning day deviations from full moon in the genus *Acropora*. (*a*) Location of the coral reefs from which coral spawning data were collected in this study. The map was created using the *leaflet* function of R v. 3.5.1. (*b*) Histogram of spawning day deviation from full moon. Solid and dashed lines with numbers show the mean and mode values, respectively. Data for all seven reefs were combined. (Online version in colour.)

We obtained information on the spawning days of *Acropora* species from seven reefs in Australia and Japan, where field observations of coral spawning have been conducted for more than 5 years (electronic supplementary material, tables S1 and S2). Four of these reefs are located in the Great Barrier Reef (Heron Island, Lizard Island, Magnetic Island and Orpheus Island) and three reefs are located in the southwest of Japan (Sekisei Lagoon, Aka Island and Otsuki; figure 1*a*). To select a single variable to represent the timing of spawning for a focal year, we focused on the date at which spawning was observed for the first time in each of the years (hereafter referred to as ‘first spawning’) and calculated the deviation between first spawning and the full moon of that month for each year and each reef (electronic supplementary material, table S1 and figure S1).

### (b) Environmental variables

We selected four environmental parameters that are reportedly related to the timing of spawning: SST [10], solar insolation [7,8], wind speed [9,10] and rate of precipitation [6] (electronic supplementary material, table S3). The change in SST between days (ΔSST) was also used as a variable, as a previous study has reported that the rate of ΔSST is highly correlated with the spawning month [10]. In total, we used five variables in our model. Daily SST (°C) values were obtained from NOAA High-resolution Blended Analysis at a 0.25° spatial resolution [17]. The global daily 1° × 1° gridded Adjusted All-Sky Surface Spectral Shortwave Down Flux (hereafter, simply referred to as ‘solar flux’) data were obtained from NASA's Clouds and the Earth's Radiant Energy System Synoptic (CERES-SYN1 deg) Edition 4A, used as a measure of solar insolation. Solar fluxes (W m^−2^) are surface solar irradiances in the shortwave region (wavelength between 0.1754 and 4 µm) [18]. The daily means of 10 m above surface wind speed (m s^−1^) and rate of precipitation (mm h^−1^) at a 0.25° spatial resolution were derived from the Tropical Rainfall Measuring Mission's (TRMM) Microwave Imager (TMI) by the NASA Earth Sciences Program and were recorded using Remote Sensing Systems. These daily environmental parameters were obtained for the years 2003–2014 and used in subsequent analyses (electronic supplementary material, table S3). The mean values of each variable were calculated for four different time ranges: (1) −1 to −30, (2) −31 to −60, (3) −61 to −90 and (4) −91 to −120 days after the full moon of the month of the first spawning (DAFM) for each year (see electronic supplementary material, figure S1 for details). These time ranges were selected so as to cover the late stage of gametogenesis in *Acropora* corals [2,14], allowing us to examine in which range the environmental factors well explained the spawning day deviation. Monthly means of parameters (SST, ΔSST, solar flux, wind speed and rate of precipitation) were standardized to *Z*-score values (a mean of zero and a standard deviation of one) for each time range.

### (c) Statistical analysis

To identify the factors affecting the night of *Acropora* coral spawning, we constructed linear mixed models (LMMs) with a Gaussian error structure, including *Z*-scores of the five assessed environmental parameters as explanatory variables and deviation from the full moon (by the DAFM) for each year as the response variable. No multi-collinearity between the environmental variables was found using variance inflation factors [19] (i.e. all five variables had variance inflation factors less than 3), and thus all variables were included in full models. The difference in reefs was included in the models as a random intercept. LMMs that included all experimental parameters (full models) were generated for each of the four different time ranges. To select the best-fit models (best LMMs) for each time range, all possible pairs of models were compared based on the Akaike information criterion (AIC) and the models with lowest AICs were selected. The data from the selected best-fit models were visualized for analytical use to examine the normality and homoscedasticity of the residuals (electronic supplementary material, figures S2–S5). Subsequently, we calculated two types of *R*^2^-values for the selected best-fit models: marginal *R*^2^ $\left(R_{m}^{2}\right)$, which indicated the variance explained in the response variable by fixed effects, and conditional *R^2^* $\left(R_{c}^{2}\right)$, which indicated the variance explained by both fixed and random effects [20]. We also calculated the proportional change in variance at the reef level (PCV_[Reef]_) for the selected models to evaluate how the addition of fixed effects to the intercept model affected the variance component at the reef level [20]. Positive and negative PCV_[Reef]_ values indicate that the variance at the reef level has decreased and increased, respectively. All analyses were performed using R v. 3.5.1 [21].

## 3. Results

Observational records of the spawning day on the seven examined reefs are summarized in electronic supplementary material, table S1 and histograms for these variables are presented in figure 1*b* (for total observations) and electronic supplementary material, figure S6 (for each reef region). Deviation from the full moon ranged from −3 (for Sekisei Lagoon in 2005) to +13 DAFM (for Otsuki in 2003), the mean and mode values were +3.83 and +5, respectively (solid and dashed vertical lines, respectively, in figure 1*b*). The spawning day deviation varied across reefs, with the mean deviation in Otsuki being highest among all seven reefs (+7.167 DAFM) and that in Sekisei Lagoon being lowest (+0.8571).

**Figure 2.**
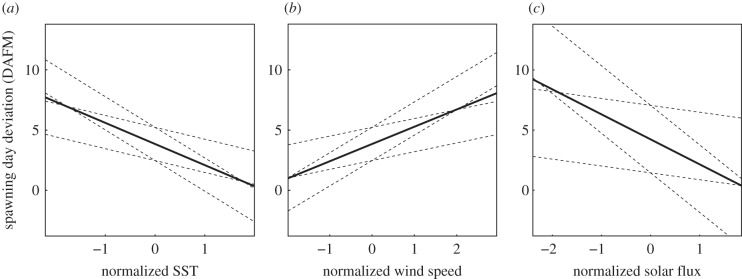
The relationships between environmental parameters and spawning day deviations from full moon estimated using best linear mixed models. Solid and dashed lines represent the regressions based on the intercepts and slopes calculated from estimated partial coefficients (solid lines) and its upper and lower 95% confidence intervals (dashed lines) under the best-fit model (see table 1 for details). Plots of spawning day deviation against standardized environmental parameters, i.e. (*a*) SSTs of time range (1), (*b*) wind speed of time range (1) and (*c*) solar flux of time range (4), are shown.

Table 1 summarizes the best LMMs selected based on the AIC values for different time ranges. The best LMMs for three of the four time ranges included the monthly mean SSTs, which had negative coefficients in all selected models. This indicated that when the monthly SST was low, the night of spawning was significantly delayed (i.e. it occurred later relative to the full moon; figure 2*a* and electronic supplementary material, figure S7). Wind speed was selected in the models for time ranges (1) and (2) and was found to positively contribute to the models (table 1; figure 2*b* and electronic supplementary material, figure S7), although the contribution was less clear in the models for time range (2) (table 1). Moreover, solar flux was selected in the best model for time range (4) and had a positive coefficient (table 1 and figure 2*c*; electronic supplementary material, figure S7). When we assessed the difference in the AIC values between the selected best model and the null model (ΔAIC), the best model of time range (1) showed the highest ΔAIC value among the four time ranges. The fixed effects of the best model of time range (1) explained 43.86% of the variance in the response variable $\left(R_{m}^{2}=43.86\text{\%}\right)$, and when considering a random effect (reef) in addition to the fixed effects, 67.26% of the variance was explained $\left(R_{c}^{2}=67.26\text{\%}\right)$. Moreover, the residual of the model fitted well with a normal distribution (electronic supplementary material, figure S2). The PCV_[reef]_ values of the best-fit models for all time ranges were positive, which indicated that the inclusion of selected environmental parameters reduced the variance component at the reef level. A summary of the statistical results of the full models for the four different time ranges is shown in electronic supplementary material, table S4.

**Table 1.** Summary of the best-fit linear mixed models (LMMs) having lowest AIC value for each of four time ranges. The results of full models were summarized in electronic supplementary material, table S4 and the diagnostic plots of these models were showed in electronic supplementary material, figure S2–S5. CI: confidence interval; VC: variance components; AIC: Akaike Information Criterion; ΔAIC: the difference of AIC between the best-fit model and the intercept model; $R_{m}^{2}$: marginal *R*^2^; $R_{c}^{2}$: conditional *R*^2^. AIC values were calculated using maximum likelihood (ML) and other parameters were generated from restricted maximum likelihood (REML).

time range	selected best models based on AIC			
(1)	**fixed effects**	***β*_coefficent_**	**lower 95% CI**	**upper 95% CI**
	** **(intercept)	3.84	2.47	5.23
	SST	−1.76	−2.55	−0.99
	wind speed	1.43	0.73	2.11
	**random effect**	**VC**		
	Reef	2.69		
	AIC	236.01		
	ΔAIC	32.11		
	$R_{m}^{2}$ (%)	43.86		
	$R_{c}^{2}$ (%)	67.26		
	PCV_[Reef]_ (%)	29.06		
(2)	**fixed effects**	***β*_coefficent_**	**lower 95% CI**	**upper 95% CI**
	(intercept)	3.94	2.29	5.59
	SST	−1.88	−2.89	−0.85
	wind speed	0.77	−0.16	1.63
	**random effect**	**VC**		
	Reef	3.87		
	AIC	258.36		
	ΔAIC	9.75		
	$R_{m}^{2}$ (%)	27.1		
	$R_{c}^{2}$ (%)	56.15		
	PCV_[Reef]_ (%)	11.66		
(3)	**fixed effect**	***β*_coefficent_**	**lower 95% CI**	**upper 95% CI**
	(intercept)	3.99	2.54	5.47
	SST	−1.52	−2.64	−0.41
	**random effect**	**VC**		
	Reef	2.77		
	AIC	263.15		
	ΔAIC	4.96		
	$R_{m}^{2}$ (%)	19.4		
	$R_{c}^{2}$ (%)	42.51		
	PCV_[Reef]_ (%)	4.12		
(4)	**fixed effect**	***β*_coefficent_**	**lower 95%CI**	**upper 95% CI**
	(intercept)	4.23	1.44	7.05
	solar flux	−2.08	−3.29	−0.57
	**random effect**	**VC**		
	Reef	11.8		
	AIC	262.89		
	ΔAIC	5.22		
	$R_{m}^{2}$ (%)	19.84		
	$R_{c}^{2}$ (%)	74.03		
	PCV_[Reef]_ (%)	13.07

## 4. Discussion

Although *Acropora* corals spawn with high levels of synchrony, the night of spawning relative to the full moon phase varies inter-annually, suggesting some interplay between environmental and endogenous (developmental and physiological) factors. Although this study was not designed to establish a causal relationship between environmental parameters and spawning events, we did use statistical measures to explain inter-annual variation in the spawning days. Our results showed that high SST and low wind speed were clearly correlated with earlier spawning events, where the mean SST and wind speed explained approximately 50% of the deviation from a full moon. A weak correlation was detected between the solar flux four months prior to a full moon and the spawning days.

Correlations between SST and spawning day have been shown in several field observations [13,14,16] and an empirical study [22], all of which reported that high seawater temperature accelerated the spawning day in *Acropora* corals, which is consistent with our results. SST potentially affects the day of spawning in two ways, namely, the progression of gamete maturation and the proximate trigger for spawning events. In corals, temperature influences gametogenesis and the reproductive cycles, and thus functions as a determinant for the spawning season [2]. The eggs in *Acropora* species become pigmented (matured) approximately three weeks prior to spawning, and spermatids appear in testes four to six weeks prior to spawning [2]. These periods correspond to time ranges (1) and (2) in the present study, in which SST had a significant effect, suggesting that high SSTs accelerate the development of gametes and subsequently the day of spawning. Several studies have proposed that hormone pathways and G protein-coupled receptors are involved in gamete release [11,23–25], which awaits further studies in light of the role of SST.

Keith *et al*. [10] found that wind speeds were weakly correlated with the month of spawning in *Acropora* spp. in the Indo-Pacific, such that spawning is more probable when wind speeds are at intermediate levels [10]. Our results also indicated that high winds in the period immediately prior to spawning might delay the night of spawning. One plausible advantage of synchronous spawning is to maximize the probability of fertilization [26–28], which high wind speed and subsequent high wave action can negate, by increasing the dispersal of gametes before fertilization, in addition to the formation of white caps that can damage naked coral embryos floating at the sea surface [29]. Corals may sense water movements and regulate their spawning behaviours to avoid periods of high wave energy. In addition, strong winds leading high waves and surface currents may change the mixed layer depth as well as sunlight reflectivity, and thus affect seawater temperature. Interaction of these factors will be a subject of future empirical studies.

Solar flux is related to the energy acquisition of coral via the photosynthesis of symbiotic algae, which may accelerate the maturation of gametes and spawning days. Another possibility is that increasing solar flux at time range (4) affected the subsequent increase in SST at time ranges (1) and (2) and indirectly accelerated the spawning days. In our study, the SST was found to lag the solar flux cycle at all the seven reefs examined (electronic supplementary material, figure S8). The difference in two fluctuating factors (SST and solar flux) may account for the effects of these factors at different time ranges.

Overall, our data are consistent with the hypothesis that *Acropora* have the capacity to fine-tune the timing of spawning, by adjusting their development and physiology in response to the environmental factors, potentially leading to increases in reproductive success and post-fertilization survival rates. Recent climate change has been shown to have significant impacts on coral reef ecosystems [30–32], e.g. rising SST due to global warming may cause the mass bleaching of corals [33,34] and is likely to go along with unpredictable effects on environmental factors. The bleaching can lead to detrimental effects on coral reproduction such as decreasing number of gonads in polyp, reduction of gravid colonies and impaired sperm motility [35–37], and the negative effects on the reproduction may persist for several years [38]. Moreover, a recent study has reported that the spawning synchrony of *Acropora eurystoma* has been attenuated in the Red Sea, which might result in reducing the probability of fertilization success [39]. This study provides a basic framework for further data-driven studies to understand the responses of corals to environmental changes as well as the underlying mechanisms that regulate their reproductive cycles.

## Supplementary Material

Supplementary figures

## Supplementary Material

Table S1

## Supplementary Material

Table S2

## Supplementary Material

Table S3

## Supplementary Material

Table S4

## Data accessibility

Data are provided as electronic supplementary material.

## References

[1] Harrison PL, Babcock RC, Bull GD, Oliver JK, Wallace CC, Willis BL 1984 Mass spawning in tropical reef corals. Science 223, 1186–1189. (10.1126/science.223.4641.1186) 17742935

[2] Babcock RC, Bull GD, Harrison PL, Heyward AJ, Oliver JK, Wallace CC, Willis BL 1986 Synchronous spawnings of 105 scleractinian coral species on the Great Barrier Reef. Mar. Biol. 90, 379–394. (10.1007/BF00428562)

[3] Hayashibara T, Shimoike K, Kimura T, Hosaka S, Heyward A, Harrison P, Kudo K, Omori M 1993 Patterns of coral spawning at Akajima Island, Okinawa, Japan. Mar. Ecol. Prog. Ser. 101, 253–262. (10.3354/meps101253)

[4] Willis BL, Babcock RC, Harrison PL, Oliver TK 1985 Patterns in the mass spawning of corals on the Great Barrier Reef from 1981 to 1984. Proc. 5th. Int. Coral Reef Congr. 4, 343–348.

[5] Oliver JK, Babcock RC, Harrison PL, Willis BL 1988 Geographic extent of mass coral spawning: clues to ultimate causal factors. Proc. 6th. Int. Coral Reef Symp. 2, 803–810.

[6] Mendes JM, Woodley JD 2002 Timing of reproduction in *Montastraea annularis*: relationship to environmental variables. Mar. Ecol. Prog. Ser. 227, 241–251. (10.3354/meps227241)

[7] Penland L, Kloulechad J, Idip D, Van Woesik R 2004 Coral spawning in the western Pacific Ocean is related to solar insolation: evidence of multiple spawning events in Palau. Coral Reefs 23, 133–140. (10.1007/s00338-003-0362-x)

[8] Van Woesik R, Lacharmoise F, Köksal S 2006 Annual cycles of solar insolation predict spawning times of Caribbean corals. Ecol. Lett. 9, 390–398. (10.1111/j.1461-0248.2006.00886.x) 16623724

[9] Van Woesik R 2010 Calm before the spawn: global coral spawning patterns are explained by regional wind fields. Proc. R. Soc. B 277, 715–722. (10.1098/rspb.2009.1524) PMC2842745 19892757

[10] Keith SA et al. 2016 Coral mass spawning predicted by rapid seasonal rise in ocean temperature. Proc. R. Soc. B 283, 20160011 (10.1098/rspb.2016.0011) PMC4874704 27170709

[11] Hayashibara T, Iwao K, Omori M 2004 Induction and control of spawning in Okinawan staghorn corals. Coral Reefs 23, 406–409. (10.1007/s00338-004-0406-x)

[12] Brady AK, Hilton JD, Vize PD 2009 Coral spawn timing is a direct response to solar light cycles and is not an entrained circadian response. Coral Reefs 28, 677–680. (10.1007/s00338-009-0498-4)

[13] Shimoike K 1999 [Observation of coral spawning in Akajima Island with newly found spawning patterns]. Midoriishi 10, 29–31. [In Japanese.]

[14] Nozawa Y 2012 Annual variation in the timing of coral spawning in a high-latitude environment: influence of temperature. Biol. Bull. 222, 192–202. (10.1086/BBLv222n3p192) 22815368

[15] Lin CH, Nozawa Y 2017 Variability of spawning time (lunar day) in *Acropora* versus merulinid corals: a 7-yr record of *in situ* coral spawning in Taiwan. Coral Reefs 36, 1269–1278. (10.1007/s00338-017-1622-5)

[16] Fujiwara S, Kezuka D, Ishimizu H, Tabata S, Nojima S 2015 [Condition for mass spawning of scleractinian coral *Acropora* in the Sekisei Lagoon, Ryukyu Islands]. Bull. Jpn. Soc. Fish. Ocean. 79, 130–140. [In Japanese.]

[17] Reynolds RW, Smith TM, Liu C, Chelton DB, Casey KS, Schlax MG 2007 Daily high-resolution-blended analyses for sea surface temperature. J. Clim. 20, 5473–5496. (10.1175/2007JCLI1824.1)

[18] Rutan DA, Kato S, Doelling DR, Rose FG, Nguyen LT, Caldwell TE 2015 CERES synoptic product: methodology and validation of surface radiant flux. J. Atmos. Ocean. Technol. 32, 1121–1143. (10.1175/jtech-d-14-00165.1)

[19] Zuur AF, Ieno EN, Elphick CS 2010 A protocol for data exploration to avoid common statistical problems. Methods Ecol. Evol. 1, 3–14. (10.1111/j.2041-210X.2009.00001.x)

[20] Nakagawa S, Schielzeth H 2013 A general and simple method for obtaining R2 from generalized linear mixed-effects models. Methods Ecol. Evol. 4, 133–142. (10.1111/j.2041-210x.2012.00261.x)

[21] R Core Team. 2018 R: a language and environment for statistical computing. Vienna, Austria: R Foundation for Statistical Computing.

[22] Paxton CW, Baria MVB, Weis VM, Harii S 2016 Effect of elevated temperature on fecundity and reproductive timing in the coral *Acropora digitifera*. Zygote 24, 511–516. (10.1017/S0967199415000477) 26349407

[23] Twan W-H, Hwang J-S, Lee Y-H, Wu H-F, Tung Y-H, Chang C-F 2006 Hormones and reproduction in scleractinian corals. Comp. Biochem. Physiolosy ParA Mol. Integr. Physiol. 144, 247–253. (10.1016/j.cbpa.2006.01.011) 16488637

[24] Kaniewska P, Alon S, Karako-Lampert S, Hoegh-Guldberg O, Levy O 2015 Signaling cascades and the importance of moonlight in coral broadcast mass spawning. Elife 4, e09991 (10.7554/eLife.09991) 26668113 PMC4721961

[25] Rosenberg Y, Doniger T, Harii S, Sinniger F, Levy O 2017 Canonical and cellular pathways timing gamete release in *Acropora digitifera*, Okinawa, Japan. Mol. Ecol. 26, 2698–2710. (10.1111/mec.14062) 28214372

[26] Pierson WJ, Moskowitz L 1964 A proposed spectral form for fully developed wind seas based on the similarity theory of S. A. Kitaigorodskii. J. Geophys. Res. 69, 5181–5190. (10.1029/jz069i024p05181)

[27] Levitan DR, Fukami H, Jara J, Kline D, McGovern TM, McGhee KE, Swanson CA, Knowlton N 2004 Mechanisms of reproductive isolation among sympatric broadcast-spawning corals of the *Montastraea annularis* species complex. Evolution 58, 308–323. (10.1111/j.0014-3820.2004.tb01648.x) 15068348

[28] Baird AH, Guest JR, Willis BL 2009 Systematic and biogeographical patterns in the reproductive biology of scleractinian corals. Annu. Rev. Ecol. Evol. Syst. 40, 551–571. (10.1146/annurev.ecolsys.110308.120220)

[29] Heyward AJ, Negri AP 2012 Turbulence, cleavage, and the naked embryo: a case for coral clones. Science 335, 1064 (10.1126/science.1216055) 22383841

[30] Spalding MD, Brown BE 2015 Warm-water coral reefs and climate change. Science 350, 769–771. (10.1126/science.aad0349) 26564846

[31] Heron SF, Maynard JA, Van Hooidonk R, Eakin CM 2016 Warming trends and bleaching stress of the world's coral reefs 1985-2012. Sci. Rep. 6, 38402 (10.1038/srep38402) 27922080 PMC5138844

[32] Hughes TP et al. 2003 Climate change, human impacts, and the resilience of coral reefs. Science 301, 929–933. (10.1126/science.1085046) 12920289

[33] Hughes TP et al. 2017 Global warming and recurrent mass bleaching of corals. Nature 543, 373–377. (10.1038/nature21707) 28300113

[34] Hughes TP et al. 2018 Spatial and temporal patterns of mass bleaching of corals in the Anthropocene. Science 359, 80–83. (10.1126/science.aan8048) 29302011

[35] Baird AH, Marshall PA 2002 Mortality, growth and reproduction in scleractinian corals following bleaching on the Great Barrier Reef. Mar. Ecol. Prog. Ser. 237, 133–141. (10.3354/meps237133)

[36] Mendes JM, Woodley JD 2002 Effect of the 1995–1996 bleaching event on polyp tissue depth, growth, reproduction and skeltal band formation in *Montastraea annularis*. Mar. Ecol. Progr. Ser. 235, 93–102. (10.3354/meps235093)

[37] Hagedorn M, Carter VL, Lager C, Camperio Ciani JF, Dygert AN, Schleiger RD, Henley EM 2016 Potential bleaching effects on coral reproduction. Reprod. Fertil. Dev. 28, 1061–1071. (10.1071/RD15526) 41382394

[38] Levitan DR, Boudreau W, Jara J, Knowlton N 2014 Long-term reduced spawning in *Orbicella* coral species due to temperature stress. Mar. Ecol. Prog. Ser. 515, 1–10. (10.3354/meps11063)

[39] Shlesinger T, Loya Y 2019 Breakdown in spawning synchrony: a silent threat to coral persistence. Science 365, 1002–1007. (10.1126/science.aax0110) 31488683

