# Supplementary material for: Environmental factors explain spawning day deviation from full moon in the scleractinian coral Acropora
Source: Biol Lett. 2020 Jan 22;16(1):20190760. doi: 10.1098/rsbl.2019.0760 (PMC7013481; doi:10.1098/rsbl.2019.0760)
Supplement: Supplementary figures [file rsbl20190760supp1.pdf]

**Supplementary figures:**

**Environmental factors explain spawning day deviation from full moon in the scleractinian coral *Acropora***

Yusuke Sakai, Masayuki Hatta, Seishiro Furukawa, Masakado Kawata, Naoto Ueno, Shinichiro Maruyama

**Figure S1. A schematic diagram of data processing for statistical analyses.** Mean environmental parameters were calculated for each year and each reef, based on time ranges 1 to 4.

**Figure S2. Diagnostic plots of the final linear mixed model for time range (1)**. The model includes spawning day deviation from the full moon as the response variable and mean sea surface temperature (SST) and wind speed as the explanatory variables. The difference in reef region was included as a random effect. (A) Normal quantile–quantile plots; (B) Distribution histograms of residuals; (C) Plot of residuals against predicted values; (D) Plot of predicted values against response variables.

**Figure S3. Diagnostic plots of the final linear mixed model for time range (2)**. The model includes spawning day deviation from the full moon as the response variable and mean sea surface temperature (SST) and wind speed as the explanatory variables. Refer to the legend for Fig. S2.

**Figure S4. Diagnostic plots of the final linear mixed model for time range (3)**. The model includes spawning day deviation from the full moon as the response variable and mean sea surface temperature (SST) as the explanatory variable. Refer to the legend for Fig. S2.

**Figure S5. Diagnostic plots of the final linear mixed model for time range (4)**. The model includes spawning day deviation from full moon as the response variable and mean solar flux as the explanatory variable. Refer to the legend for Fig. S2.

**Figure S6. Histograms of spawning day deviation from the full moon for each of the seven examined reefs.**

**Figure S7. Summary of the typical seasonal changes in environmental parameters [sea surface temperature (SST), wind speed, and solar flux].** The vertical straight and undulating lines represent the spawning day deviation from the full moon and the daily mean values of environmental parameters of the years showing lowest (red) or highest (blue) deviation at each reef, respectively. The environmental data were plotted from -120 DAFM.

**Fig. S8. Annual fluctuations in sea surface temperature (SST) and solar flux in 2010 and 2011 in seven reef regions.** Lines show the daily means of SST and solar flux, which were standardized to Z-score values, respectively.

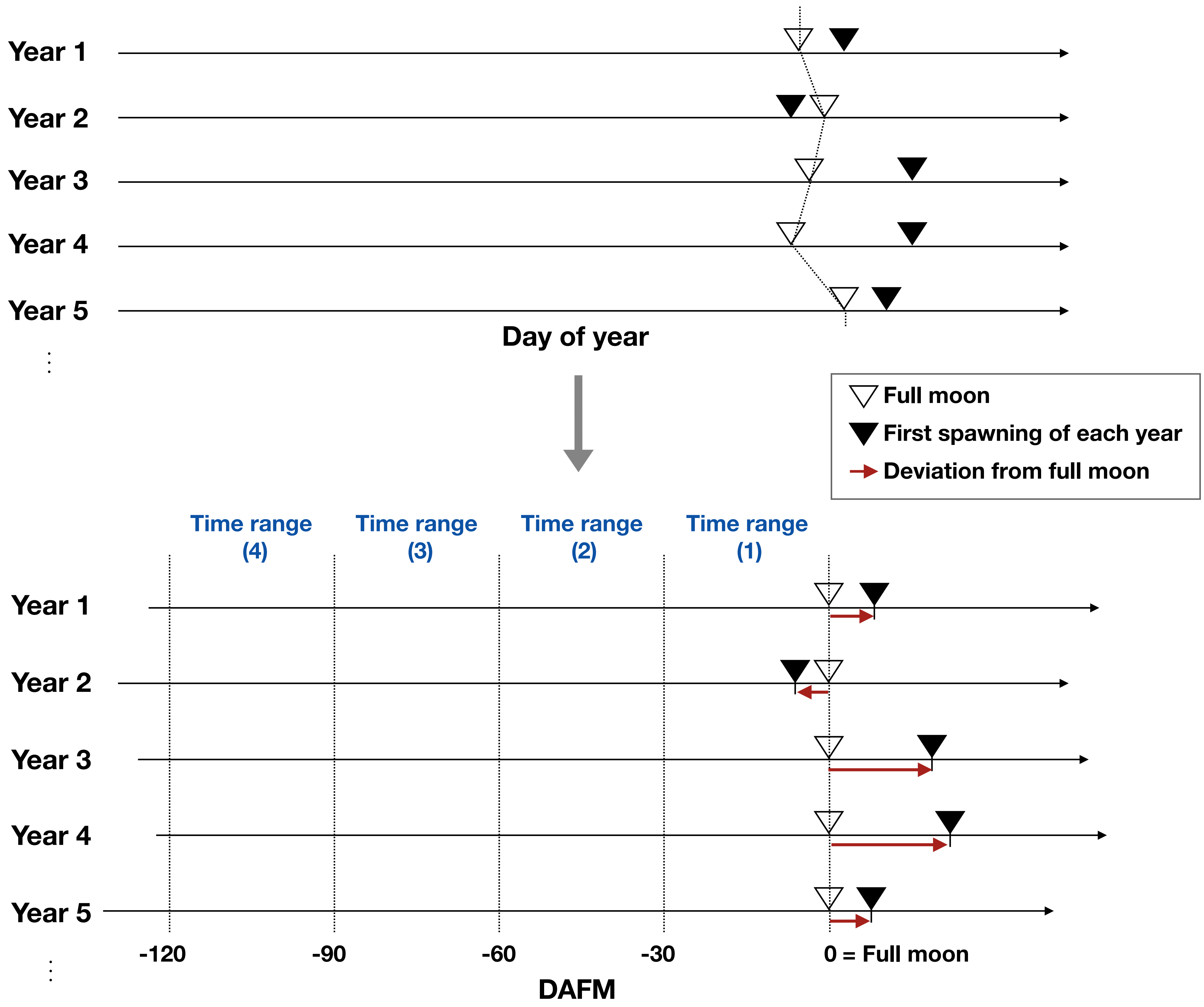

Year 1
Year 2
Year 3
Year 4
Year 5
Day of year
Full moon
First spawning of each year
Deviation from full moon
Time range (4)
Time range (3)
Time range (2)
Time range (1)
Year 1
Year 2
Year 3
Year 4
Year 5
-120
-90
-60
-30
0 = Full moon
DAFM


Figure S1

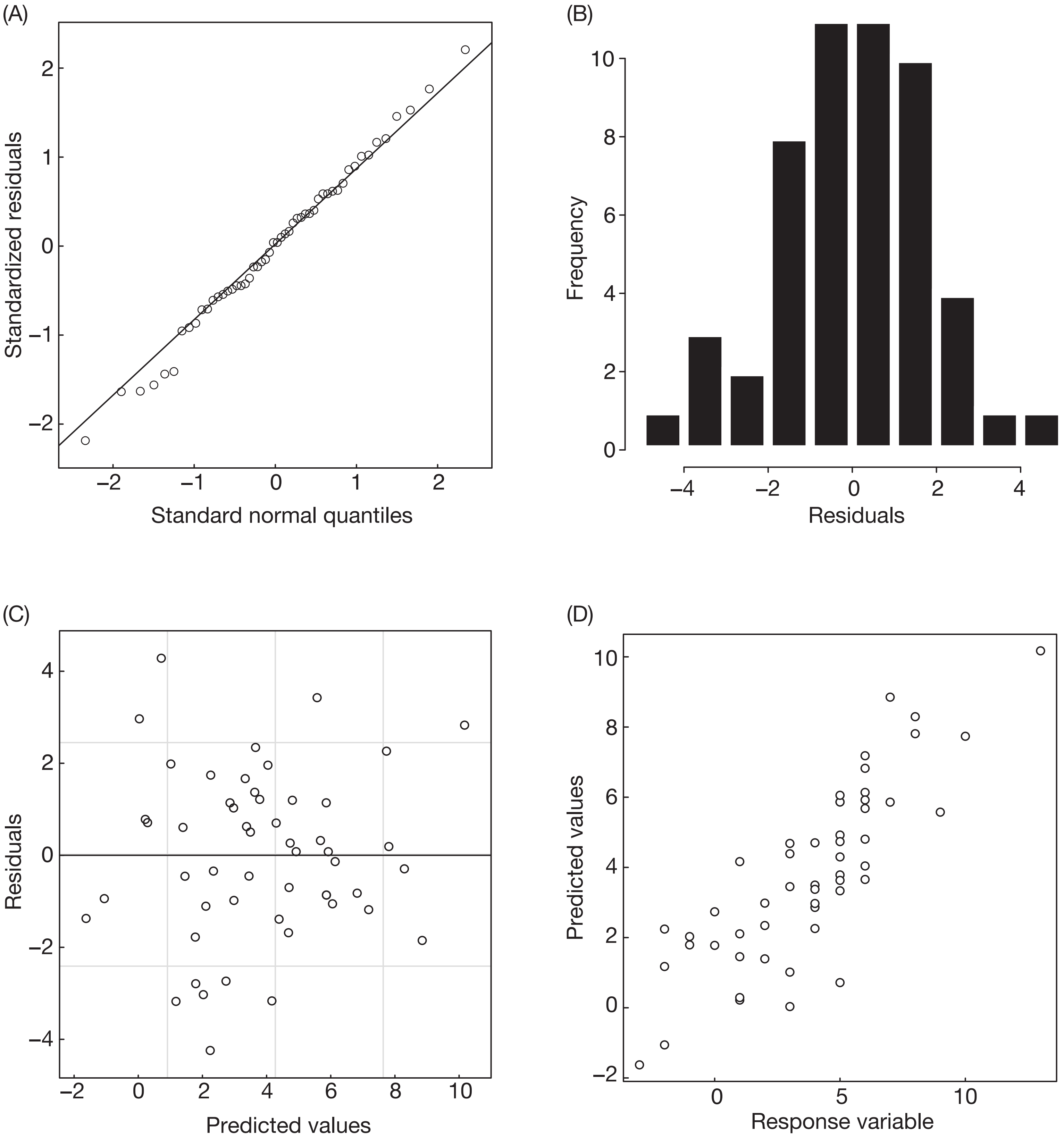
(A)
Standardized residuals
Standard normal quantiles
(B)
Frequency
Residuals
(C)
Residuals
Predicted values
(D)
Predicted values
Response variable

Figure S2

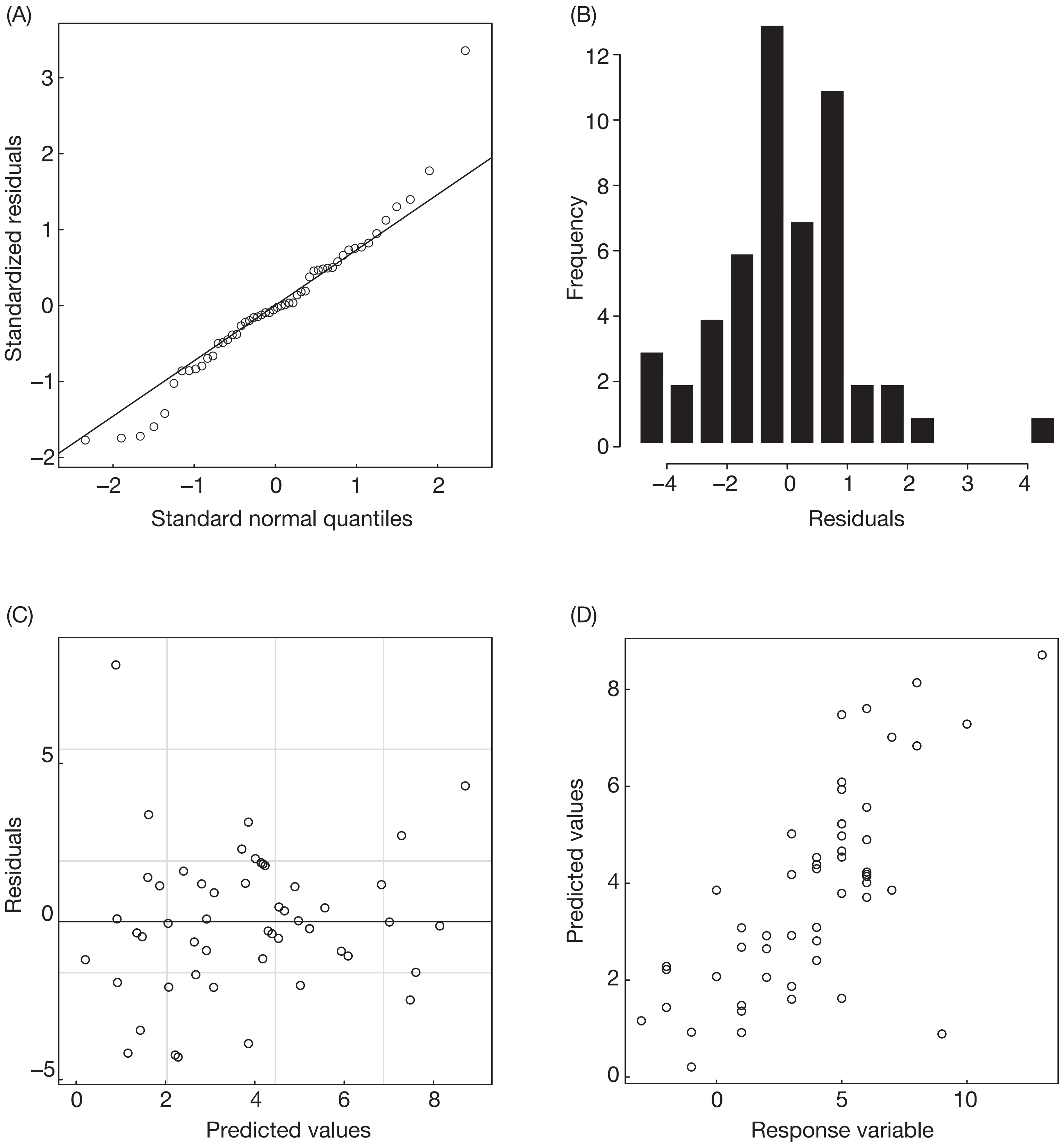
(A)
Standardized residuals
Standard normal quantiles
(B)
Frequency
Residuals
(C)
Residuals
Predicted values
(D)
Predicted values
Response variable

Figure S3

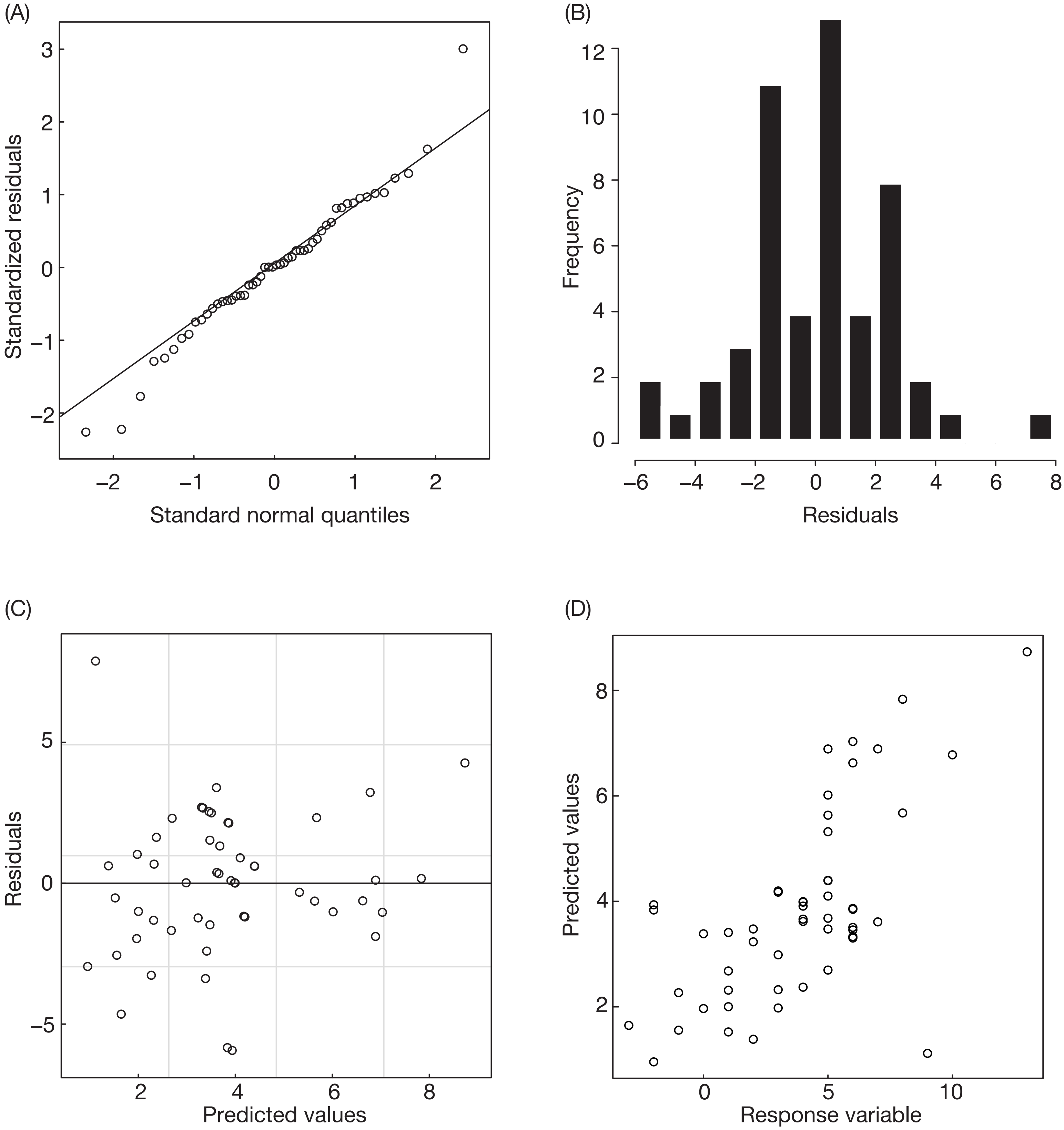
(A)
Standardized residuals
Standard normal quantiles
(B)
Frequency
Residuals
(C)
Residuals
Predicted values
(D)
Predicted values
Response variable

Figure S4

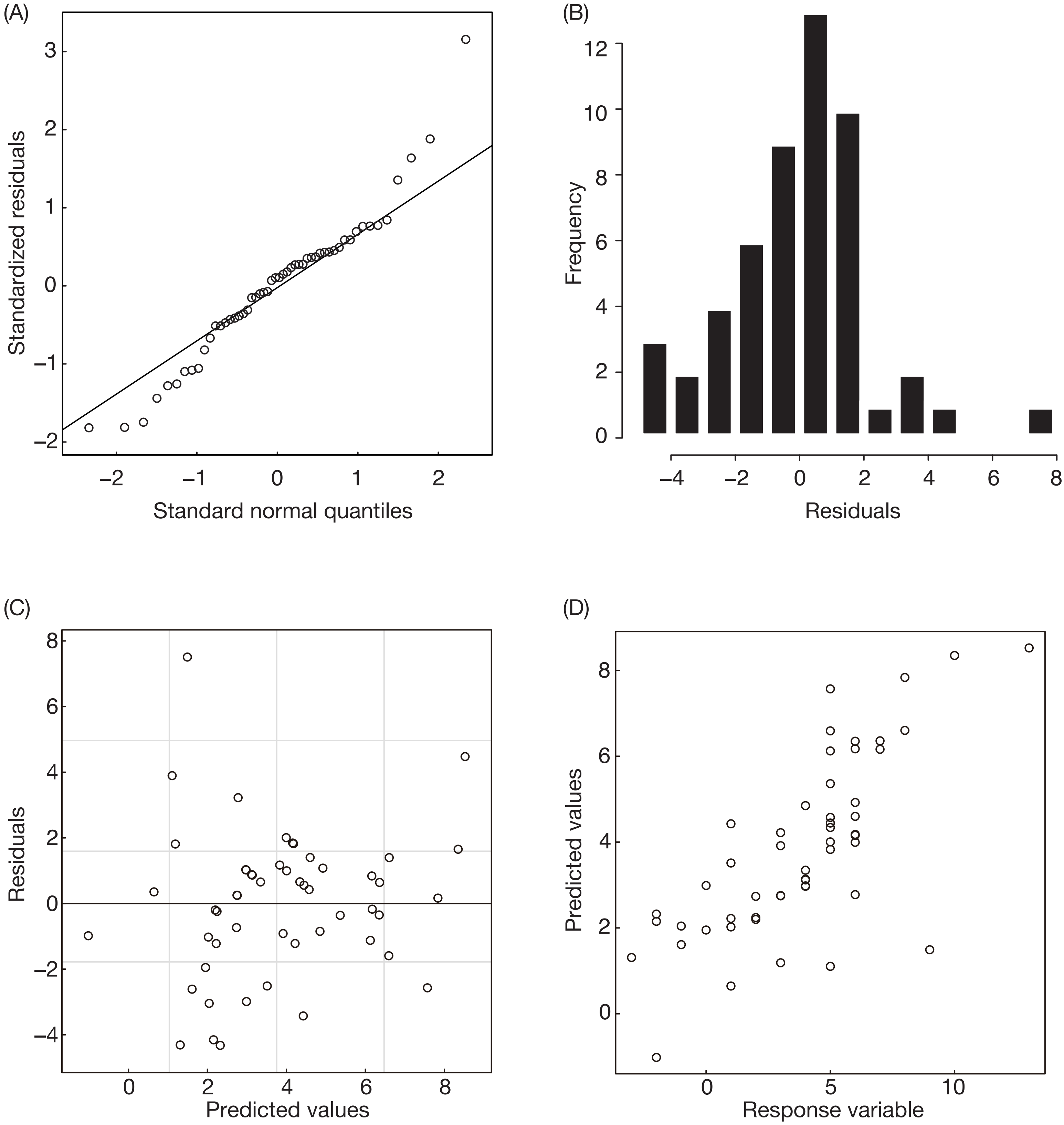
(A)
Standardized residuals
Standard normal quantiles
(B)
Frequency
Residuals
(C)
Residuals
Predicted values
(D)
Predicted values
Response variable

Figure S5

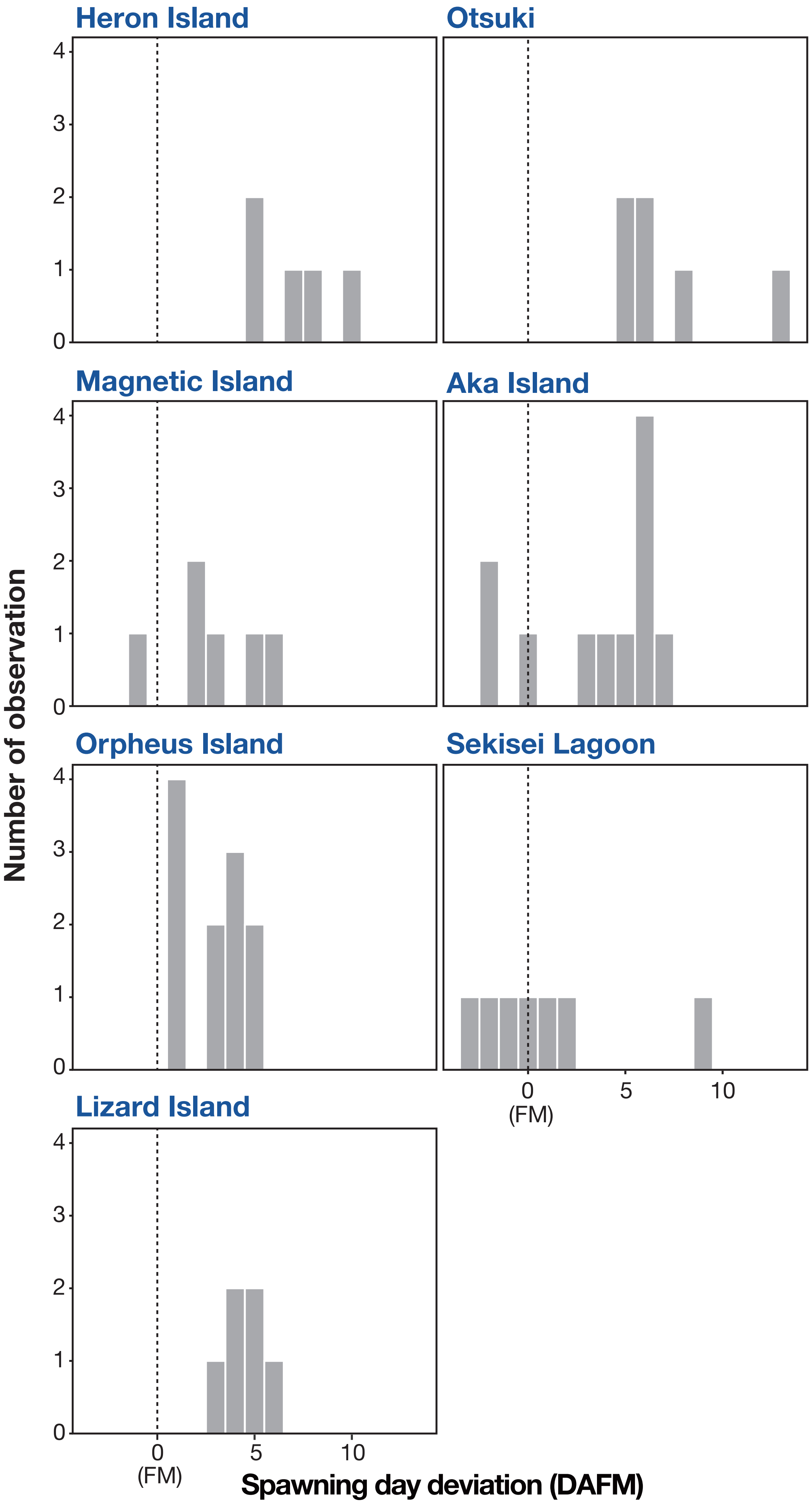
Heron Island
Otsuki
Magnetic Island
Aka Island
Orpheus Island
Sekisei Lagoon
Lizard Island
Number of observation
0
1
2
3
4
0
(FM)
5
10
Spawning day deviation (DAFM)

Figure S6

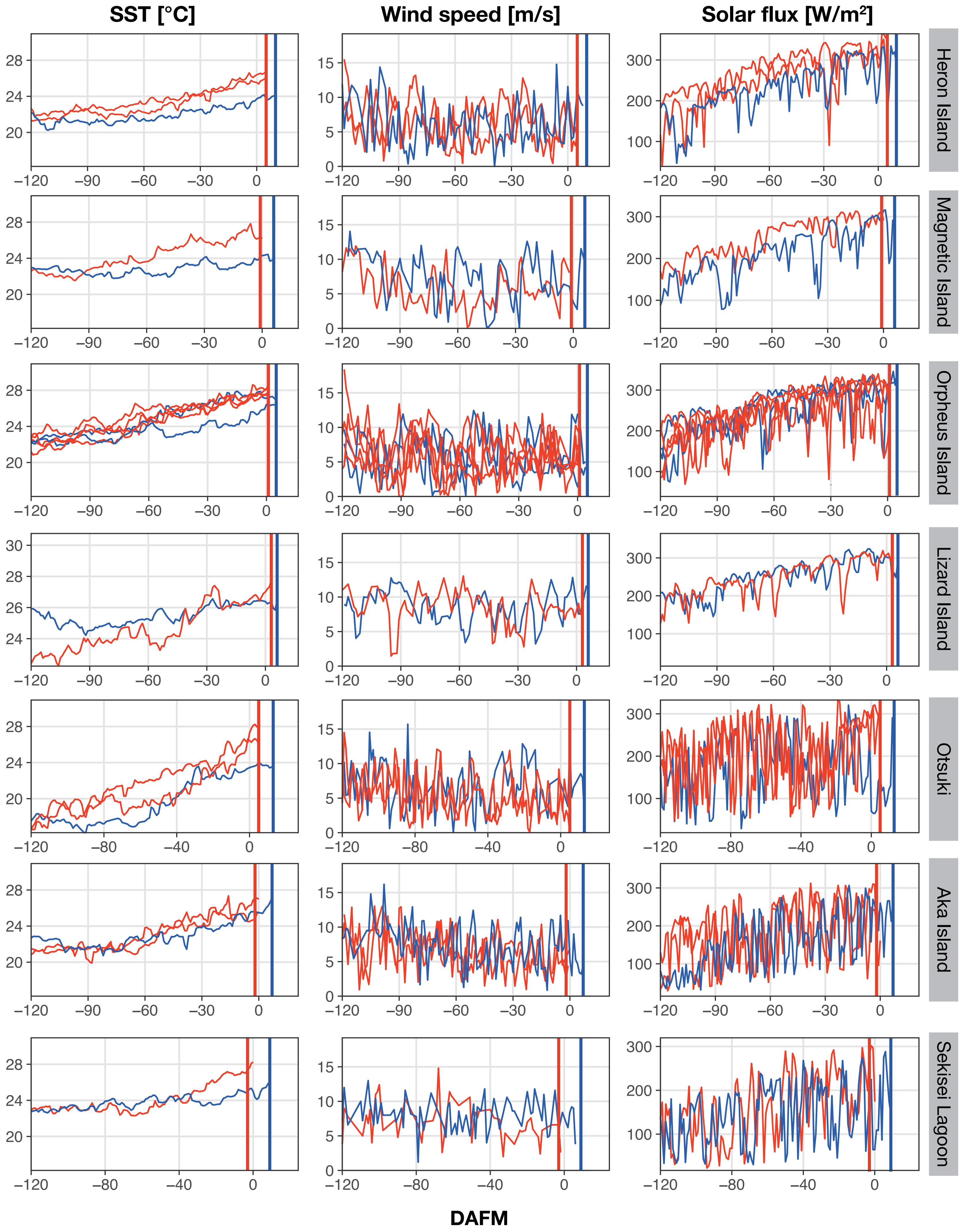
SST [°C]
Wind speed [m/s]
Solar flux [W/m²]
Heron Island
Magnetic Island
Orpheus Island
Lizard Island
Otsuki
Aka Island
Sekisei Lagoon
DAFM

Figure S7

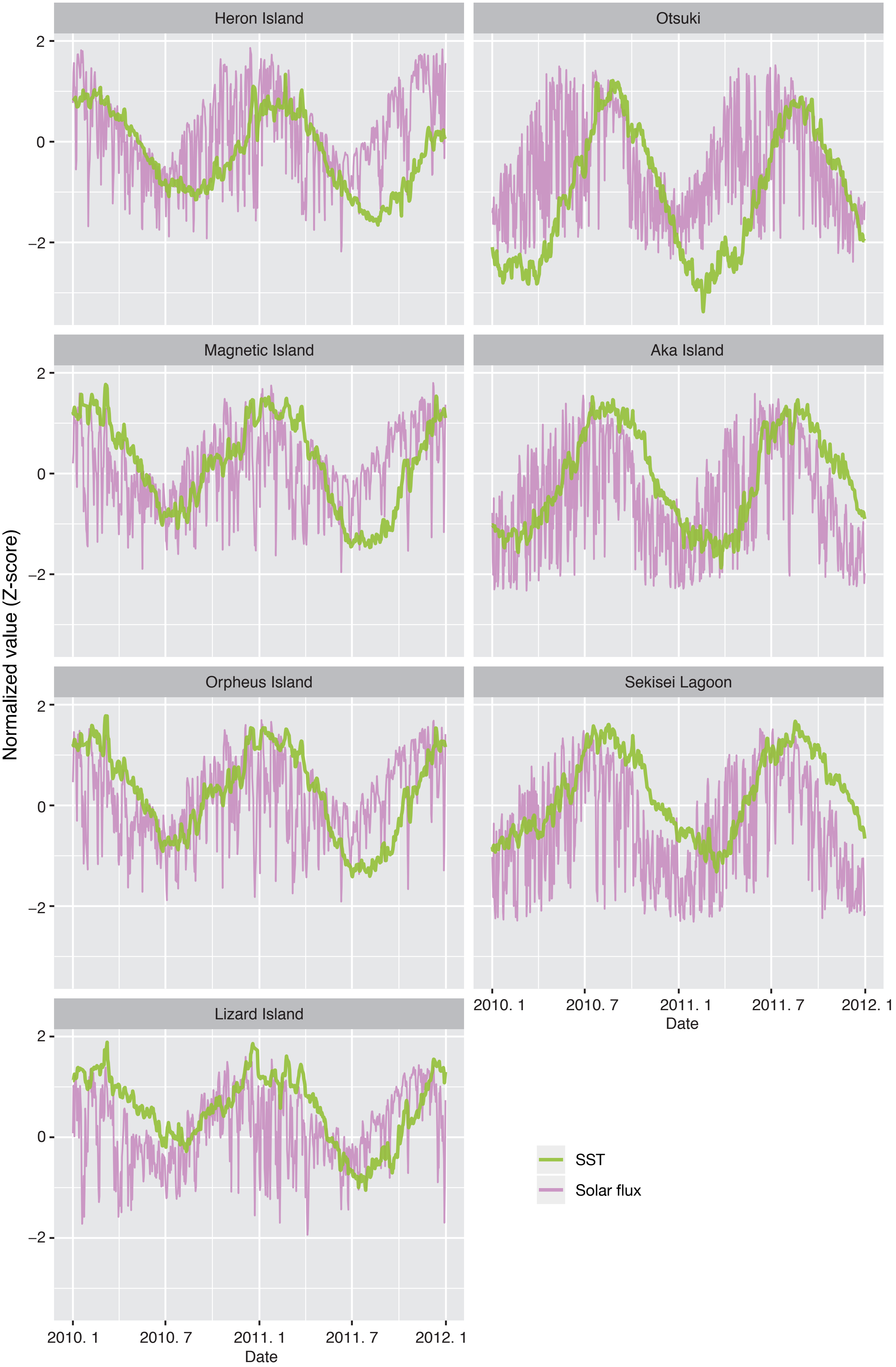

Heron Island
Otsuki
Magnetic Island
Aka Island
Orpheus Island
Sekisei Lagoon
Lizard Island
Normalized value (Z-score)
2
0
−2
2010. 1
2010. 7
2011. 1
2011. 7
2012. 1
Date
SST
Solar flux


Figure S8
